# A Novel Gelatinase from Marine *Flocculibacter collagenilyticus* SM1988: Characterization and Potential Application in Collagen Oligopeptide-Rich Hydrolysate Preparation

**DOI:** 10.3390/md20010048

**Published:** 2022-01-03

**Authors:** Jian Li, Jun-Hui Cheng, Zhao-Jie Teng, Xia Zhang, Xiu-Lan Chen, Mei-Ling Sun, Jing-Ping Wang, Yu-Zhong Zhang, Jun-Mei Ding, Xin-Min Tian, Xi-Ying Zhang

**Affiliations:** 1 Xinjiang Key Laboratory of Biological Resources and Genetic Engineering, College of Life Science and Technology, Xinjiang University, Urumqi 830046, China; fighter1216217564@163.com; 2 State Key Laboratory of Microbial Technology, Marine Biotechnology Research Center, Institute of Marine Science and Technology, Shandong University, Qingdao 250100, China; 201820299@mail.sdu.edu.cn (J.-H.C.); tengpaper@163.com (Z.-J.T.); cxl0423@sdu.edu.cn (X.-L.C.); jp1120714999@163.com (J.-P.W.); 3 Department of Molecular Biology, Qingdao Vland Biotech Inc., Qingdao 266102, China; zhangx@vlandgroup.com; 4 College of Marine Life Sciences, and Frontiers Science Center for Deep Ocean Multispheres and Earth System, Ocean University of China, Qingdao 266003, China; sunml1990@yeah.net (M.-L.S.); zhangyz@sdu.edu.cn (Y.-Z.Z.); 5 Engineering Research Center of Sustainable Development and Utilization of Biomass Energy, Ministry of Education, Yunnan Normal University, Kunming 650500, China

**Keywords:** peptidase, the MEROPS S8 family, bovine bone collagen, oligopeptides, hydrolysate

## Abstract

Although the S8 family in the MEROPS database contains many peptidases, only a few S8 peptidases have been applied in the preparation of bioactive oligopeptides. Bovine bone collagen is a good source for preparing collagen oligopeptides, but has been so far rarely applied in collagen peptide preparation. Here, we characterized a novel S8 gelatinase, Aa2_1884, from marine bacterium *Flocculibacter collagenilyticus* SM1988^T^, and evaluated its potential application in the preparation of collagen oligopeptides from bovine bone collagen. Aa2_1884 is a multimodular S8 peptidase with a distinct domain architecture from other reported peptidases. The recombinant Aa2_1884 over-expressed in *Escherichia coli* showed high activity toward gelatin and denatured collagens, but no activity toward natural collagens, indicating that Aa2_1884 is a gelatinase. To evaluate the potential of Aa2_1884 in the preparation of collagen oligopeptides from bovine bone collagen, three enzymatic hydrolysis parameters, hydrolysis temperature, hydrolysis time and enzyme-substrate ratio (E/S), were optimized by single factor experiments, and the optimal hydrolysis conditions were determined to be reaction at 60 ℃ for 3 h with an E/S of 400 U/g. Under these conditions, the hydrolysis efficiency of bovine bone collagen by Aa2_1884 reached 95.3%. The resultant hydrolysate contained 97.8% peptides, in which peptides with a molecular weight lower than 1000 Da and 500 Da accounted for 55.1% and 39.5%, respectively, indicating that the hydrolysate was rich in oligopeptides. These results indicate that Aa2_1884 likely has a promising potential application in the preparation of collagen oligopeptide-rich hydrolysate from bovine bone collagen, which may provide a feasible way for the high-value utilization of bovine bone collagen.

## 1. Introduction

Bioactive oligopeptides are referred to peptides that consist of 2–20 amino acids, which have various bioactivities [1]. In addition to being efficient amino acid sources, bioactive oligopeptides have been reported to possess many physiological functions and attractive physic properties in pharmacy (e.g., anticancer, antimicrobial, antihypertensive and anti-inflammatory activities, anticoagulant, and immunomodulatory), foods (gelling activity and emulsifying property), cosmetic (antioxidant and water holding capacity), and other functional products (foaming ability and hydrophobicity) [2,3,4,5]. In recent years, collagen oligopeptides attract more and more attention due to their various bioactive properties, such as angiotensin I converting enzyme (ACE) inhibitory activity, antioxidant activity, immunomodulatory and antimicrobial activities [6,7,8,9], and beneficial effects on human health, including improving skin health, muscle strength, and bone density [10,11,12], and reducing obesity, joint pain, and blood pressure [13,14,15]. Collagen oligopeptides have been widely applied in food, cosmetics, healthcare, and pharmaceutical industries [16,17,18].

Enzymatic hydrolysis is now the common method to prepare collagen bioactive peptides from collagen-rich animal tissues, such as skin, bones, tendons, and ligaments. Nowadays, the common enzymes for preparing collagen bioactive peptides are proteases from plants, animals, and bacteria, such as serine proteases alcalase of the MEROPS S8 family and trypsin and α-chymotrypsin of the MEROPS S1 family, aspartic protease pepsin of the MEROPS A1 family, cysteine protease papain of the MEROPS C1 family, and metalloprotease thermolysin of the MEROPS M4 family [6,19,20,21,22]. The S8 family is the second largest family of serine proteases after the S1 family [23]. In the S8 family, many members have activity on gelatin, the denatured form of collagen, and some are collagenolytic proteases, such as the thermostable protease from *Geobacillus collagenovorans* MO-1 [24], MCP-01 from *Pseudoalteromonas* sp. SM9913 [25], myroicolsin from *Myroides profundi* D25 [26], and P57 from *Photobacterium* sp. A5-7 [27]. Due to their activity on natural or denatured collagen, these S8 peptidases may have potentials in collagen oligopeptide preparation. However, only a few S8 peptidases have been used in preparing collagen oligopeptides, or their potentials have been evaluated. In addition to alcalase that are from *Bacillus* and have been used in collagen oligopeptide preparation [28], MCP-01 has also been shown to have a potential in preparing collagen bioactive peptides from codfish skin [7]. It is still necessary to identify more S8 peptidases suitable for preparing collagen bioactive peptides.

Recently, we isolated and identified a novel marine bacterium *Flocculibacter collagenilyticus* SM1988^T^ (hereafter SM1988) that has a high collagenase production [29]. According to the genome and secretome analyses of this strain, Aa2_1884 was the most abundant of the 6 secreted S8 proteases and was predicted to be a potential collagenase [29]. The aim of this study was to characterize Aa2_1884 and to evaluate its potential in preparing collagen bioactive peptides. In this study, Aa2_1884 was expressed in *Escherichia coli* and biochemically characterized. The potential of Aa2_1884 in preparing collagen oligopeptides from bovine bone collagen was further evaluated. The results indicate that Aa2_1884 is a novel multimodular gelatinase with a good potential in preparing collagen oligopeptide from bovine bone collagen.

## 2. Results and Discussion

### 2.1. Aa2_1884 Is a Novel Multimodular Peptidase of the S8 Family

**Figure 1:**
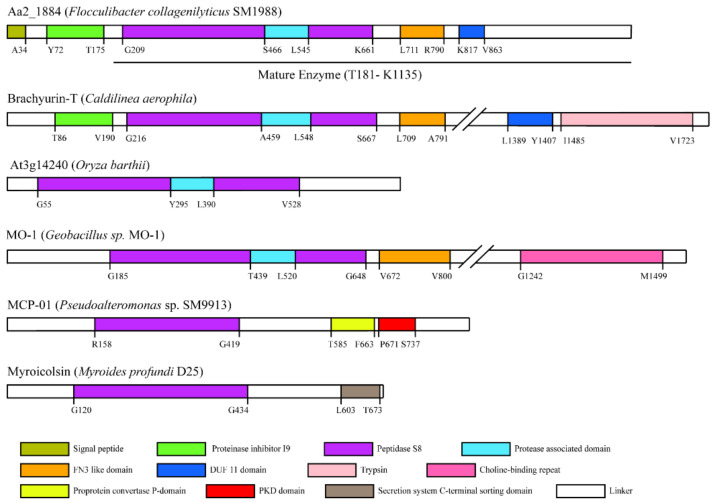
Domain architectures of Aa2_1884 and similar proteases predicted by InterPro. Marked numbers show the corresponding position of predicted domains in the amino acid sequences of the proteases.

**Figure 2:**
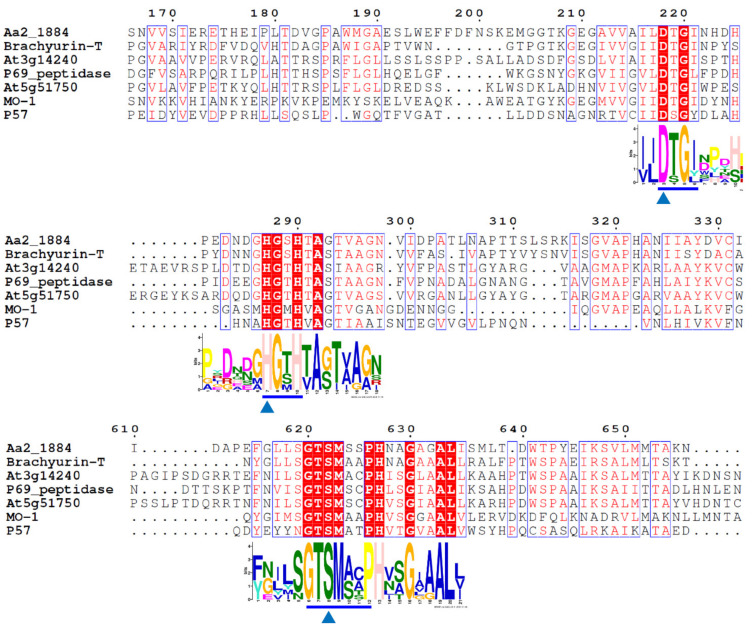
Sequence alignment of Aa2_1884 with similar proteases and reported S8 collagenases using the ClustalW program. Similar amino acid residues are boxed and shown in red, conserved amino acid residues are shown with red background. Amino acid residues constituting the catalytic triad of the MEROPS S8 family are marked with blue triangles, and motifs containing these residues are marked with blue underlines. Aa2_1884 (WP_199608745) is from *Flocculibacter collagenilyticus* SM1988, brachyurin-T (YP_005442656) from *Caldilinea aerophile*, At3g14240 (MER0006049) from *Oryza barthii*, P69 peptidase (XP_002275452) from *Vitis vinifera*, At5g51750 (XP_009789180) from *Nicotiana sylvestris*, MO-1 (BAF30978) from *Geobacillus* sp. MO-1, and P57 (KT923662) protease from *Photobacterium* sp. A5-7.

The amino acid sequence of protein Aa2_1884 (WP_199608745.1) deduced from the genome of strain SM1988 is composed of 1135 amino acid residues, containing a signal peptide with a length of 34 amino acid residues at the N terminus based on the SignalP 5.0 prediction. Aa2_1884 is annotated as an S8 family serine peptidase by BLASTP through the non-redundant protein database. InterProScan analysis indicated that, in addition to the predicted signal peptide, Aa2_1884 has five conserved domains (Figure 1), including an inhibitor I9 domain (Tyr72-Thr175, IPR010259), a peptidase S8 domain (Gly209-Lys661, IPR000209), a protease associated (PA) domain (Ser466-Leu545, IPR003137), a fibronectin type-III (FN3) domain (Leu711-Arg790, IPR041469), and a domain of unknown function (DUF11) (Lys817-Val863, IPR001434). The inhibitor I9 domain likely functions as a molecular chaperone to assist the protein folding of Aa2_1884 [30,31]. The peptidase S8 domain is the catalytic domain, containing the characteristic catalytic triad of the S8 family, namely Asp218, His287, and Ser622 (Figure 2). The PA domain is an inserted domain in the peptidase S8 domain, which has been shown to play a role in collagen binding in some S8 proteases [27,32]. FN3 domain has been found in several proteases [33,34], whose function in proteases, however, has not been revealed. DUF11 domain is also present in peptidase brachyurin-T of the S1 family from *Caldilinea aerophile* based on InterProScan prediction (Figure 1). However, its function in peptidases such as brachyurin-T and Aa2_1884 needs further study.

**Figure 3:**
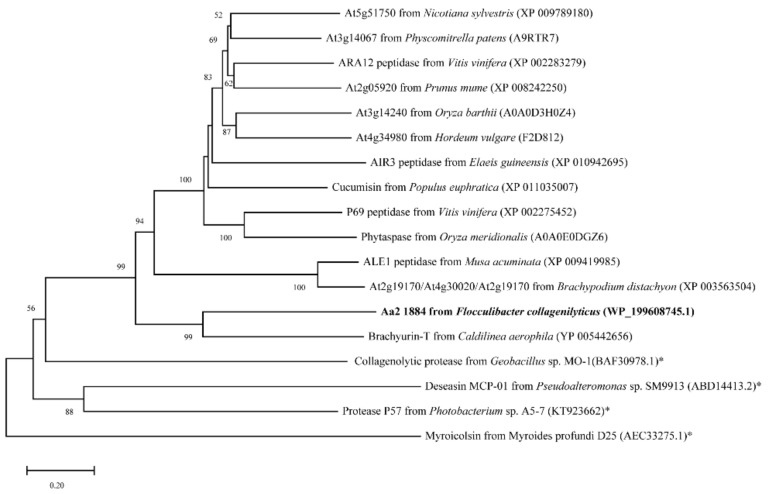
Neighbor-joining (NJ) phylogenetic tree based on amino acid sequences of Aa2_1884 and similar proteases. The tree was constructed with MEGA X. Bootstrap values (>50%) based on 1000 replicates were presented at nodes. Bar, 0.20. The reported S8 collagenases are indicated by asterisks.

Among the characterized peptidases, Aa2_1884 shares the highest sequence identity (44.05%) with brachyurin-T of the S1 family [35]. It is also most close to brachyurin-T in the phylogenetic tree (Figure 3). However, the domain architectures of these two enzymes are different. Compared to brachyurin-T, Aa2_1884 lacks the C-terminal Trypsin domain (Figure 1). Among all the characterized peptidases, none were found to have the same domain architecture as Aa2_1884 (Figure 1). These data suggest that Aa2_1884 is a novel multimodular protease of the S8 family. In addition, as shown in Figure 2, sequence alignment indicated that Aa2_1884 contains several motifs that are conserved in reported S8 collagenases, which suggests that Aa2_1884 may have collagenolytic activity.

### 2.2. Aa2_1884 Is a Gelatinase with High Activity toward Denatured Collagens

**Figure 4:**
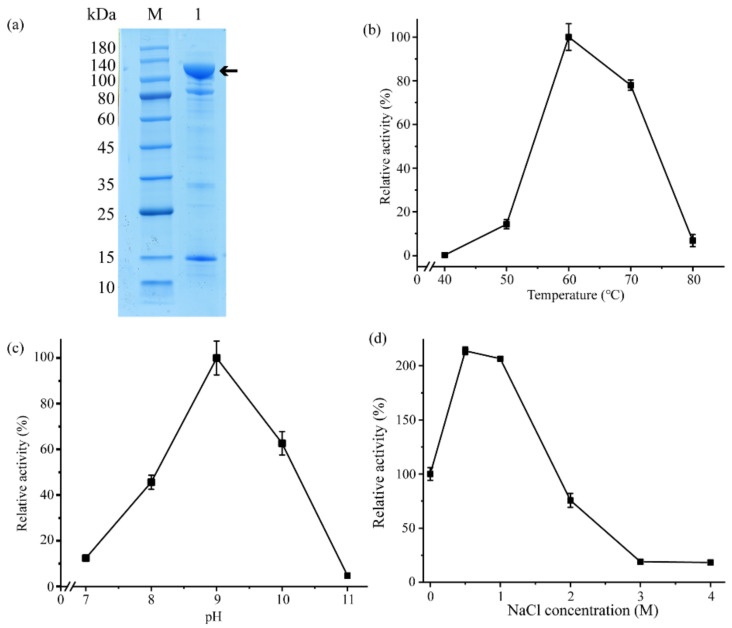
Purification and characterization of Aa2_1884: (**a**) SDS-PAGE analysis of purified Aa2_1884. Lane M, protein mass markers. Lane 1, purified Aa2_1884. The protein band of Aa2_1884 is indicated by an arrow; (**b**) Effect of temperature on the activity of Aa2_1884. The experiment was performed in Tris-HCl buffer (50 mM, pH 9.0) at 40–80 °C, and the enzyme activity at 60 °C was taken as 100%; (**c**) Effect of pH on the activity of Aa2_1884. The enzyme activity was measured at 60 °C with 40 mM Britton–Robinson buffers ranged from pH 7 to pH 11. The enzyme activity at pH 9.0 was taken as 100%; (**d**) Effect of NaCl concentration on the activity of Aa2_1884. The activity was measured at 60 °C in Tris-HCl buffer (50 mM, pH 9.0) containing different concentrations of NaCl from 0 to 4 M. The enzyme activity in the Tris-HCl buffer with 0 M NaCl was taken as 100%. In (**b**–**d**), bovine bone collagen was used as the substrate in the experiments. The graphs show data from triplicate experiments (mean ± SD).

To characterize Aa2_1884, the gene of Aa2_1884 was over-expressed in *Escherichia coli* BL21 (DE3) with the vector pET-22b (+) containing a C-terminal His tag. The recombinant Aa2_1884 protein was purified by affinity chromatography on a His Bind Ni chelating column and gel filtration chromatography on a Sephadex G200 column. Sodium dodecyl sulfate polyacrylamide gel electrophoresis (SDS-PAGE) analysis showed that the purified Aa2_1884 had an apparent molecular weight of approximately 100,000 Da (Figure 4a). The N-terminal sequence of the purified Aa2_1884 was determined to T181-D-V-G-P-A186 by N-terminal sequencing. Thus, on the basis of its N-terminal sequence and molecular weight, mature Aa2_1884 should contain 955 amino acid residues from Thr181 to Lys1135 (Figure 1). The signal peptide and the inhibitor I9 domain are cleaved off during maturation.

Sequence alignment implied that Aa2_1884 may have collagenolytic activity. Indeed, Aa2_1884 had noticeable activity towards bovine bone collagen at temperatures of 50–70 °C, with an optimal temperature at 60 °C. However, it had almost no activity towards bovine bone collagen at 40 °C (Figure 4b). At 60 °C, Aa2_1884 also had activity towards bovine tendon collagen, gelatin, and casein, but no activity toward elastin-orcein (Table 1). As collagen is denatured at temperatures more than 40 °C, these results indicate that Aa2_1884 can hydrolyze denatured collagen, but not natural collagen. Therefore, Aa2_1884 is a gelatinase, rather than a collagenase. With bovine bone collagen as the substrate, Aa2_1884 showed the highest activity at pH 9.0 (Figure 4c), indicating that it is an alkaline protease. In the buffer containing different NaCl (0–4 M), Aa2_1884 showed the highest activity at 0.5–1 M NaCl (Figure 4d). These characteristics reflect the adaptation of Aa2_1884 to the marine salty and alkaline environment.

**Figure 5:**
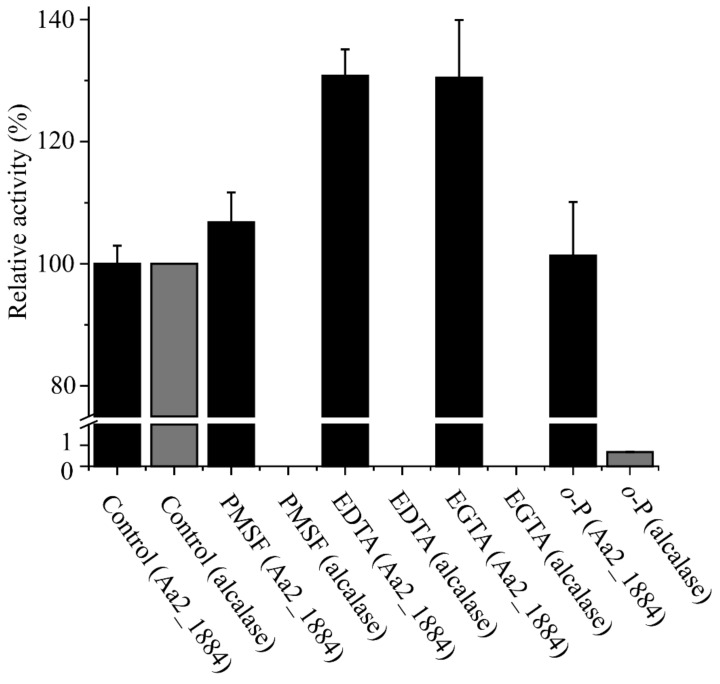
Effects of protease inhibitors on the activity of Aa2_1884. Aa2_1884 and the control enzyme alcalase were incubated at 4 ℃ for 1 h in 50 mM Tris-HCl (pH 9.0) containing 2 mM of each inhibitor, PMSF, EDTA, EGTA, or *o*-P. After incubation, the residual activity toward bovine bone collagen was measured at 60 °C, pH 9.0. The activity of Aa2_1884 without any inhibitor was taken as 100%. The graphs show data from triplicate experiments (mean ± SD).

We also analyzed the effects of metal ions and protease inhibitors on the activity of Aa2_1884. As shown in Table 2, Ca^2+^, Ba^2+^, Sr^2+^, and Mg^2+^ significantly increased the activity of Aa2_1884 towards bovine bone collagen, Zn^2+^ and Fe^2+^ severely inhibited its activity, and Ni^2+^, Co^2+^, and Mn^2+^ completely inhibited its activity (Table 2). Surprisingly, none of the four tested inhibitors, phenylmethylsulfonyl fluoride (PMSF), ethylenediamine tetraacetic acid (EDTA), ethylene glycol tetraacetic acid (EGTA), or o-phenanthroline (*o*-P), had inhibitory effect on the activity of Aa2_1884 (Figure 5), which is an unusual phenomenon for an S8 peptidase. Among these inhibitors, PSMF is a classical inhibitor to serine proteases, however, there are also some exceptions. Kexin has been reported to be resistant to PMSF [36]. Kexin is a typical S8 peptidase produced by *Saccharomyces cerevisiae*, which contains 814 amino acid residues. Different from Aa2_1884, Kexin contains only a P-proprotein domain in addition to the Peptidase S8 domain [37]. It still remains elusive why kexin is resistant to PMSF. Thus, the underlying mechanisms of kexin and Aa2_1884 to resist PMSF need further investigation.

The S8 family, the second largest family of serine peptidases, contains more than 200 peptidases in the MEROPS database (https://www.ebi.ac.uk/merops/cgi-bin/famsum?family=S8, accessed on 6 December 2021). In this family, only a small number have collagenolytic activity, but many have gelatinolytic activity. Different from those with collagenolytic activity, Aa2_1884 has no activity towards native collagen. On the other hand, although Aa2_1884 has gelatinolytic activity, it has a distinct domain architecture and is resistant to PMSF, compared to those with gelatinolytic activity in the S8 family. Therefore, Aa2_1884 is a new gelatinase of the S8 family. The S8 family includes diverse peptidases produced by bacteria, archaea, and eukaryotes from various environments, and most are secreted endopeptidases. Therefore, both the temperature and pH optima of the S8 peptidases are in a wide range due to the adaptation of the peptidases to their respective environments [38]. For example, assays of subtilisins from *Bacillus* species are typically performed at pH 8.2–8.6 and 25 °C [39]. The pH optima of the S8 peptidases from archaea are usually in the range of 7.5–10.7, and their temperature optima are in a wide range of 55–115 °C [40]. The pH optima of the S8 collagenases from bacteria are within the range of 7.1–9.3, and their temperature optima are usually 50–60 °C [24,26,41,42]. The optimal temperature and pH of Aa2_1884 are 60 °C and pH 9.0, which fall in the optimal temperature and pH ranges of the S8 peptidases.

### 2.3. Aa2_1884 Shows High Hydrolytic Efficiency on Bovine Bone Collagen

**Figure 6:**
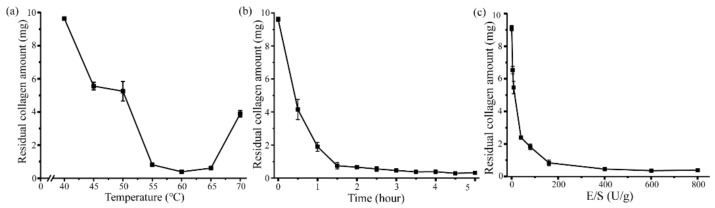
Effects of temperature, time and E/S on the hydrolysis efficiency of bovine bone collagen: (**a**) Effect of hydrolysis temperature determined at the hydrolysis time of 3 h and the hydrolysis E/S of 400 U/g; (**b**) Effect of hydrolysis time determined at the hydrolysis temperature of 60 °C and the hydrolysis E/S of 400 U/g; (**c**) Effect of hydrolysis E/S determined at the hydrolysis temperature of 60 °C and the hydrolysis time of 3 h. The graphs show data from triplicate experiments (mean ± SD).

As Aa2_1884 had high activity towards bovine bone collagen at 60 °C (Figure 4b), it may have a potential in preparing collagen bioactive peptides from bovine bone collagen. Thus, attempts were made to prepare peptides from bovine bone collagen with Aa2_1884 as a tool. To determine the optimal hydrolysis conditions, three enzymatic hydrolysis parameters were optimized by single factor experiments, including hydrolysis temperature, hydrolysis time and enzyme-substrate ratio (E/S). On the basis of the residual amount of collagen, the appropriate hydrolysis temperature and time of Aa2_1884 were determined to be 55–65 °C (Figure 6a) and ≥3 h (Figure 6b), respectively. When the E/S was more than 400 U/g, no more obvious decrease in the amount of residual collagen was detected (Figure 6c). Hence, considering the hydrolysis efficiency and economic benefit, the optimal conditions of Aa2_1884 for the hydrolysis of bovine bone collagen on the laboratory scale were determined to be reaction at 60 °C for 3 h with an E/S ratio of 400 U/g. Under these hydrolysis conditions, the maximum hydrolytic efficiency of bovine bone collagen reached 95.3 ± 0.3%, indicating that Aa2_1884 is a good enzyme for the hydrolysis of bovine bone collagen. We then prepared bovine bone collagen hydrolysate with Aa2_1884 under the determined hydrolysis conditions.

### 2.4. The Collagen Hydrolysate Prepared with Aa2_1884 Is Rich in Collagen Oligopeptides

**Figure 7:**
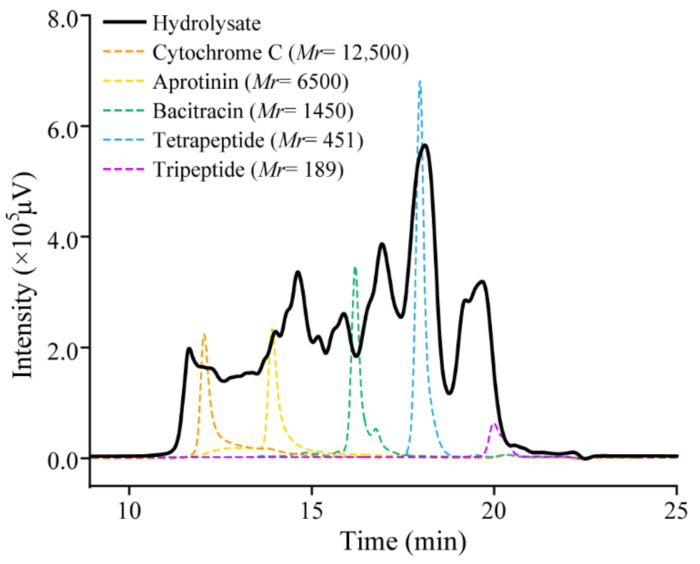
Size exclusion chromatography analysis of the molecular weight distribution of peptides in the hydrolysate. The hydrolysate dissolved in deionized water was analyzed by HPLC with a TSK gel G2000 SWXL column.

To evaluate the quality of the prepared hydrolysate, we analyzed the contents of amino acids and peptides, amino acid composition, and molecular weight distribution of peptides in the hydrolysate. According to the ninhydrin method, there were 2.2 ± 0.1% free amino acids and 97.8 ± 0.1% peptides in the hydrolysate. Analysis of the composition of free amino acids in the hydrolysate by automatic amino acid analyzer also showed that there was only a small amount of free amino acids in the hydrolysate (Table 3). Thus, the hydrolysate is rich in peptides. In the hydrolysate, glycine is the most abundant (17.2%), followed by proline (10.1%). In addition, there were 1.0% hydroxylysine and 8.2% hydroxyproline in the peptides in the hydrolysate (Table 3). As hydroxylysine and hydroxyproline are two unique amino acids in collagen, our results indicated that the hydrolysate is rich in collagen peptides. The molecular weight distribution of peptides in the hydrolysate was analyzed by high performance liquid chromatography (HPLC) (Figure 7). The results showed that peptides with a molecular weight lower than 3000 Da, 1000 Da, and 500 Da accounted for approximately 71.6 ± 0.2%, 55.1 ± 0.2%, and 39.5 ± 0.2%, respectively (Table 4), indicating that the hydrolysate is rich in collagen oligopeptides. 

### 2.5. Antioxidant Activity of Bovine Bone Collagen Hydrolysate

**Figure 8:**
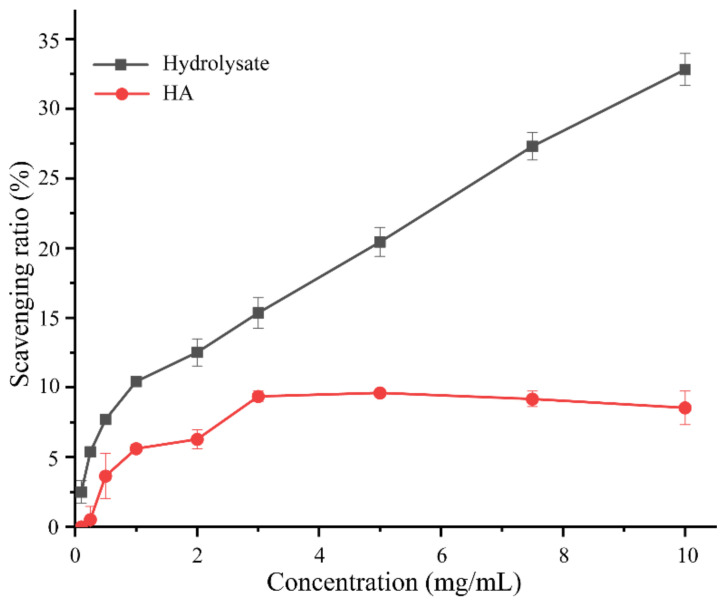
Antioxidant activity towards DPPH• of the hydrolysate and hyaluronic acid (HA).

The antioxidant activity of the hydrolysate was further evaluated by measuring its free radical scavenging activity towards 1,1-diphenyl-2-picryl-hydrazyl radical (DPPH•) with hyaluronic acid (HA) as a control. The scavenging ratio of the hydrolysate to DPPH• increased with the hydrolysate concentration, which reached 32.8 ± 1.1% at the concentration of 10 mg/mL (Figure 8). In addition, as shown in Figure 8, the DPPH• scavenging ratio of the hydrolysate was obviously higher than that of HA, especially at high concentrations. A comparison of the DPPH• scavenging ratio of the hydrolysate with those of some reported collagen hydrolysates are shown in Table 5. The differences in the DPPH• scavenging ratios among the hydrolysates are likely attributed to the differences in the collagen sources, the preparation methods, and the enzymes used.

As oligopeptides from various proteins have been demonstrated as beneficial compounds for skin protection or against diseases such as hypertension, hypercholesterolemia, and atherosclerosis, oligopeptides have been widely prepared from a variety of proteins, including proteins from various plant fruits and seeds, and proteins from skins and meats of various marine and terrestrial animals [4,47,48]. Protein hydrolysates containing oligopeptides have been prepared with both commercial and non-commercial proteases. For example, a loach protein hydrolysate prepared with papain contained approximately 30% oligopeptides with a molecular weight lower than 500 Da, and exhibited good hydroxyl radical scavenging and antioxidant activities [49]. A salmon skin hydrolysate prepared with alcalase and papain contained approximately 90% oligopeptides with a molecular weight lower than 1000 Da, and showed the ACE inhibitory effect from different fractions collected by reversed-phase HPLC [19]. A 1301 Da peptide from the cod fish skin hydrolysate prepared with pepsin, trypsin, and α-chymotrypsin exhibited potent ACE inhibitory and antioxidant activities [20]. A shrimp hydrolysate prepared with the crude enzyme from *Bacillus* sp. SM98011 contained approximately 41% oligopeptides with molecular mass lower than 3000 Da, and exhibited good hydroxyl radical scavenger and antioxidant activities [50]. A codfish skin hydrolysate prepared with the collagenolytic protease MCP-01 from *Pseudoalteromonas* sp. SM9913 contained 60% oligopeptides with a molecular weight lower than 1000 Da, and exhibited good hydroxyl radical scavenging activity and promoted an effect on cell viability of human dermal fibroblasts [7]. A bovine bone collagen hydrolysate prepared with the thermolysin-like protease A69 from *Anoxybacillus caldiproteolyticus* 1A02591 contained 21.1% oligopeptides with a molecular weight lower than 1000 Da, and exhibited good moisture-retention ability and antioxidant activity [43]. The hydrolysate prepared from Bigeye tuna skin collagen contained peptides with molecular weights of 300–425 Da and had DPPH• scavenging activity [51]. It has been demonstrated that di-/tripeptides can be absorbed in their intact forms in human intestine without further hydrolysis [5]. Thus, protein hydrolysates containing more oligo-peptides with a molecular weight of <1000 Da or even <500 Da are preferred in cosmetics, functional food, and nutraceuticals [52].

Collagen used in collagen oligopeptides preparation have been extracted from the bones and skins of various animals, such as fish skins and bones [7,44,53], and goat skin [54]. Bovine bone is a by-product of beef processing industry and its annual production is huge due to the large number of global slaughtered cattle. Bovine bone is rich in collagen and therefore, is a cheap and good source for collagen preparation. However, bovine bone collagen has rarely been used in collagen oligopeptide preparation to our knowledge. In this study, we used Aa2_1884 to prepare collagen oligopeptides from bovine bone collagen. Aa2_1884 showed a high hydrolysis efficiency (95%) on bovine bone collagen and the resultant hydrolysate contained a high proportion of collagen oligopeptides, 55.1% peptides with a molecular weight lower than 1000 Da, and 39.5% peptides with a molecular weight lower than 500 Da. These data suggest that Aa2_1884 has a promising potential in preparing collagen oligopeptides from bovine bone collagen, which may provide a feasible way for the high-value utilization of bovine bone collagen.

## 3. Materials and Methods

### 3.1. Materials

Bovine tendon collagen was purchased from Worthington (Lakewood, NJ, USA), and bovine bone collagen from Kinry Biotech Co., Ltd. (Jinan, China). Casein (from bovine milk), gelatin (from cold water fish skin), elastin-orcein, EGTA, *o*-P, aprotinin, and cytochrome C were purchased from Sigma (St. Louis, MO, USA). Alcalase was purchased from Vazyme Biotech Co., Ltd. (Nanjing, China). PMSF was purchased from BBI (Shanghai, China). EDTA was purchased from HUSHI (Shanghai, China). Bacitracin was purchased from Aladdin (Shanghai, China). Tripeptide GGG and tetrapeptide GGYR were synthesized by Qiangyao Biotechnology Co., Ltd. (Shanghai, China). DPPH• was purchased from Tokyo Chemical Industry (Tokyo, Japan). HA was purchased from Shandong Freda Bioeng Co., Ltd. (Jinan, China). Other chemicals were of analytical grade and commercially available.

### 3.2. Sequence Analysis

The domains of Aa2_1884 (WP_199608745) from *Flocculibacter collagenilyticus* SM1988 (CP05988) and of the other proteases shown in Figure 1 were predicted by InterPro (https://www.ebi.ac.uk/interpro/; 6 December 2021) [55]. The signal peptide of Aa2_1884 was predicted by the SignalP 5.0 server (https://services.healthtech.dtu.dk/service.php?SignalP-5.0; 6 December 2021) [56]. For sequence alignment, previously reported MCP-01 (ABD14413) from *Pseudoalteromonas* sp. SM9913, the collagenolytic protease (BAF30978) from *Geobacillus* sp. MO-1, myroicolsin (AEC33275) from *Myroides profundi* D25, and P57 (KT923662) from *Photobacterium* sp. A5-7 were selected to align with Aa2_1884 by ClustalW with bootstrap of 1000 [57]. The sequence alignment was displayed using ESPript 3.0 [58]. The conserved sites were predicted by MEME (https://meme-suite.org/meme/; 6 December 2021) [59]. The phylogenetic tree was constructed via MEGA X [60].

### 3.3. Protein Expression and Purification

The genome DNA of strain SM1988 was extracted with bacterial genomic DNA isolation kit (BioTeke, Beijing, China) according to the manufacturer’s instructions. The gene sequence of Aa2_1884 was amplified by PCR using the genome DNA of strain SM1988 with primers 1884-F (5′-AAGAAGGAGATATACATATGATGAAAATAGAACATAGT-3′) and 1884-R (5′-TGGTGGTGGTGGTGCTCGAGTTTATTGTCACACGTGGTT-3′). The primers were synthesized by Tsingke Biotechnology Co., Ltd. (Qingdao, China). The PCR product was then cloned into vector pET-22b (+) (Vazyme) with a C-terminal His tag. The constructed plasmid carrying the gene sequence of Aa2_1884 was verified by sequencing and then transformed into Fe2 BL21 (DE3). Recombinant *E. coli* cells were cultured in Lysogeny broth (LB) medium with 100 µg/mL ampicillin at 37 °C, 180 rpm to an OD_600_ of 0.8–1.0. Then, 0.2 mM isopropyl β-D-1-thiogalactopyranoside (IPTG) was added, and the cells were further incubated at 15 °C, 120 rpm for 5 days [26]. After incubation, cells were lysed by a high-pressure cracker and centrifuged at 4656× *g*, 4 °C for 1 h, and the supernatant was collected. The recombinant Aa2_1884 protein was extracted from the supernatant by an His binding Ni chelating column, and then purified on a Sephadex G200 gel filtration column (GE, Boston, MA, USA) using fast protein liquid chromatography (FPLC) on AKTA purifier (GE, Boston, MA, USA) [61]. The purified Aa2_1884 was analyzed by 12.5% SDS-PAGE. Protein concentration was determined by a BCA protein assay kit (Thermo, Waltham, MA, USA) with bovine serum albumin (BSA) as the standard according to the manufacturer’s instructions.

### 3.4. Enzyme Assay

The activities of Aa2_1884 toward bovine bone collagen, bovine tendon collagen, and gelatin at 60 °C were measured by the method provided by Worthington Biochemical Co. (Lakewood, NJ, USA) [26]. For collagen, a mixture of 5 mg substrate and 1 mL enzyme solution was incubated at 60 °C in Tris-HCl buffer for 0.5 h with continuous stirring. For gelatin, 100 μL enzyme solution was incubated with 100 μL of 2% (*w/v*) gelatin at 60 °C for 10 min. The reaction was stopped by the addition of 10 μL of 1.25 M trichloroacetic acid. The released amino acids were quantified using the colorimetric ninhydrin method [62] with L-leucine as the standard. One unit of enzyme activity was defined as the amount of enzyme that released 1 nmol of L-leucine per hour from collagen or gelatin [26]. The caseinolytic activity was determined at 60 °C using the method described by He et al. [63]. A reaction mixture containing 100 μL enzyme solution and 100 μL of 2% (*w/v*) casein was incubated at 60 °C for 10 min, and then the reaction was terminated by 200 μL trichloroacetic acid (0.4 M). The mixture was centrifuged at 17,935× *g* for 10 min, and 100 μL of the supernatant was incubated with 500 μL of sodium carbonate solution (0.4 M) and 100 μL of the Folin-phenol reagent at 40 °C for 20 min. After the reaction, the OD_660_ of the mixture was measured. One unit of enzyme activity was defined as the amount of enzyme that liberated 1 mg tyrosine per minute [63]. The elastolytic activity at 60 °C was determined using the method described by Chen [64]. A mixture of 250 μL enzyme solution and 5 mg elastin-orcein was incubated at 60 °C for 1 h. After the reaction, the residual elastin-orcein was removed by centrifugation. The OD_590_ of the supernatant was recorded. One unit of enzyme activity was defined as the amount of enzyme that caused an increase of 0.01 in OD_590_ per minute [64].

### 3.5. Enzyme Characterization

The optimal temperature was determined by measuring the activity of Aa2_1884 toward bovine bone collagen in Tris-HCl buffer (50 mM, pH 9.0) at 40, 50, 60, 70, and 80 °C. The optimal pH was determined by measuring the activity of Aa2_1884 toward bovine bone collagen at 60 °C in 40 mM Britton–Robinson buffers from pH 7.0 to pH 11.0. The effect of NaCl concentration on the activity of Aa2_1884 was determined by measuring the activity of Aa2_1884 toward bovine bone collagen in Tris-HCl buffer (50 mM, pH 9.0) containing NaCl of different concentrations (0–4 M) at 60 °C. To evaluate the effect of metal ions (Li^+^, K^+^, Ca^2+^, Mg^2+^, Cu^2+^, Ni^2+^, Mn^2+^, Ba^2+^, Fe^2+^, Zn^2+^, Co^2+^, Sn^2+^, Sr^2+^) on the enzymatic activity, Aa2_1884 was incubated in Tris-HCl buffer (50 mM, pH 9.0) containing each metal ion (2 mM or 4 mM) at 4 °C for 1 h, and the enzymatic activity toward bovine bone collagen was then measured at 60 °C. For the inhibitory experiment, Aa2_1884 and alcalase were incubated at 4 °C for 1 h with 2 mM of an inhibitor, PMSF, EDTA, EGTA, or *o*-P. After incubation, the residue activity toward bovine bone collagen was measured at 60 °C and pH 9.0.

### 3.6. Optimization of the Enzymatic Hydrolysis Parameters

Three parameters, hydrolysis temperature, hydrolysis time, and E/S, which influence the efficiency of the enzymatic hydrolysis, were optimized via single-factor experiments, in which enzymatic hydrolysis of 10 mg bovine bone collagen in 1 mL 50 mM Tris-HCl (pH 9.0) was performed at 180 rpm in a shaking bath. Each parameter was determined under the optimum conditions of the other two parameters. To determine the hydrolysis temperature, the enzymatic hydrolysis was performed at different hydrolysis temperature (40, 45, 50, 55, 60, 65, 70 °C). To determine the hydrolysis time, the enzymatic hydrolysis was performed for different time (0.5, 1, 1.5, 2, 2.5, 3, 3.5, 4, 4.5, 5 h). To determine the E/S, the enzymatic hydrolysis was performed with different E/S (0, 4, 8, 40, 80, 160, 400, 600, 800 U/g collagen). After the hydrolysis, the reaction system was heated at 90 °C for 15 min to terminate the reaction, and then centrifuged at 4 °C for 20 min. The precipitate was freeze-dried and weighted, which was taken as the residual amount of collagen.

### 3.7. Preparation and Evaluation of Collagen Hydrolysate

To prepare peptides from bovine bone collagen, 10 mg bovine bone collagen was hydrolyzed under the determined parameters (at 60 °C for 3 h with an E/S ratio of 400 U/g). After hydrolysis, the reaction system was heated at 90 °C for 15 min and then centrifuged at 4 °C for 20 min. The supernatant was collected, freeze-dried, and weighted, which was the prepared hydrolysate.

Ten milligrams of the hydrolysate were dissolved in 1 mL deionized water. With L-leucine as the standard, the content of free amino acids in the hydrolysate solution was determined by the ninhydrin method [62]. The content of peptides in the hydrolysate was calculated by subtracting the content of free amino acids from that of the hydrolysate in the solution [43]. The compositions of free and total amino acids of the hydrolysate were analyzed by using an amino acid analyzer HITACHI 835 (Tokyo, Japan). The molecular mass distribution of peptides in the hydrolysates were analyzed by the method described by [7]. Briefly, the hydrolysate was dissolved with deionized water, and then analyzed by HPLC (LC-20AD, SHIMADZU, Tokyo, Japan) equipped with a TSK gel G2000 SWXL column (300 × 7.8 mm; range, <150,000 Da; void volume, 5.7 mL; Tosoh, Japan) that was eluted with the buffer containing 45% acetonitrile and 1% trifluoroacetic acid in deionized water at a flow rate of 0.5 mL/min HPLC under 220 nm monitoring. The calibration standards for molecular mass were tripeptide Gly-Gly-Gly (GGG, *Mr* 189), tetrapeptide Gly-Gly-Tyr-Arg (GGYR, *Mr* 451), bacitracin (*Mr* 1422), aprotinin (*Mr* 6511), and cytochrome C (*Mr* 12400). Based on the calibration standards, the chromatogram of the hydrolysate was separated into several fractions (<500 Da, 500–1000 Da, 1000–3000 Da, 3000–5000 Da, 5000–10,000 Da, and >10,000 Da), and the content of each fraction was determined by its relative peak area.

### 3.8. Analysis of the Antioxidant Activity of the Collagen Hydrolysate

The antioxidant activity of the prepared hydrolysate was analyzed by measuring its free radical scavenging activity towards 1,1-diphenyl-2-picryl-hydrazyl radical (DPPH•) according to the method described by Sun [65]. HA was used as a positive control due to its widespread application in scavenging free radical. To determine the DPPH• scavenging activity, 1 mL hydrolysate samples in incremental concentrations (0.1, 0.25, 0.5, 1, 2, 3, 5, 7.5, 10 mg/mL) were reacted with 2 mL of 100 µM DPPH• (dissolved in ethanol solution) for 40 min at room temperature (25 °C) in dark, and then the absorbance of the reaction solution was detected at 525 nm. DPPH solution was replaced with ethanol solution to obtain the result of background of sample, and the hydrolysate sample was replaced with water to obtain the result of blank control.

The free radical scavenging activity (*D*) was calculated as follows:

(1) $$D\left(\%\right)\;=\;\left[1-\left(A_{i}-A_{j}\right)/A_{0}\right]\;*\;100$$

where $A_{i}$ was the absorbance of the sample, $A_{j}$ was the background absorbance of the sample, and $A_{0}$ was the absorbance of the blank control.

## 4. Conclusions

As only a few peptidases of the S8 family so far have been applied in the preparation of bioactive oligopeptides, there is still a need to develop more S8 serine peptidases with potentials in bioactive oligopeptide preparation. In this study, the peptidase Aa2_1884 from marine bacterium *Flocculibacter collagenilyticus* SM1988^T^ was demonstrated to be a novel multimodular gelatinase of the S8 family, which has high activity towards gelatin and denatured collagens. Moreover, under the optimized enzymolysis conditions, Aa2_1884 has a high hydrolysis efficiency (95%) on bovine bone collagen. The prepared hydrolysate is rich in collagen oligopeptides and has antioxidant activity. The results in this study suggest that Aa2_1884 has a promising potential application in preparing collagen oligopeptides from bovine bone collagen. The collagen hydrolysate prepared with Aa2_1884 may have good bioactivities due to its high oligopeptide content as well as good antioxidant activity, which awaits further investigation.

## Figures and Tables

**Table 1:** The substrate specificity of Aa2_1884 ^$^.

Substrate	Enzymatic Activity (U/mL)
Bovine bone collagen	801.15 ± 46.45
Bovine tendon collagen	761.27 ± 43.41
Gelatin	834.00 ± 61.39
Casein	51.75 ± 1.85
Elastin-orcein	–

^$^ The activities of Aa2_1884 towards different substrates were measured in Tris-HCl (50 mM, pH 9.0) at 60 °C. The data represent the mean ± SD of three experimental repeats.

**Table 2:** Effects of metal ions on the activity of Aa2_1884 ^a^.

Metal Ion	Relative Activity (%)		Metal Ion	Relative Activity (%)	
	2 mM	4 mM		2 mM	4 mM
Control	100	100	K^+^	108.62 ± 8.24	116.60 ± 4.18
Ca^2+^	263.67 ± 7.06	251.90 ± 10.14	Sn^2+^	97.01 ± 0.12	100.32 ± 4.40
Ba^2+^	250.54 ± 11.34	276.80 ± 10.14	Zn^2+^	56.50 ± 2.99	40.69 ± 1.33
Sr^2+^	245.91 ± 6.45	210.87 ± 29.18	Fe^2+^	43.73 ± 4.78	5.05 ± 2.04
Mg^2+^	217.58 ± 7.25	217.25 ± 32.74	Ni^2+^	– ^b^	–
Li^+^	111.41 ± 4.40	116.60 ± 4.18	Co^2+^	–	–
Cu^2+^	100.32 ± 2.96	122.83 ± 2.43	Mn^2+^	–	–

^a^ The activity of Aa2_1884 was measured in Tris-HCl (50 mM, pH 9.0) at 60 °C with bovine bone collagen as the substrate. The activity (657.37 U/mL) without any metal ion was used as a control (100%). The data represent the mean ± SD of three experimental repeats. ^b^ “–” means that enzyme activity was not detectable.

**Table 3:** Composition and content of free and total amino acids in the hydrolysate ^a^.

Amino Acid	Total Amino Acids (g/100 g)	Free Amino Acids (g/100 g)
Ala	6.87 ± 0.05	0.07 ± 0.02
Arg	7.16 ± 0.06	
Asp	3.39 ± 0.03	0.10 ± 0.02
Cit	0.07 ± 0.03	
Cys	0.56 ± 0.04	0.12 ± 0.01
Glu	4.04 ± 0.03	
Gly	17.21 ± 0.12	0.02 ± 0.01
His	0.67 ± 0.02	
Ile	1.54 ± 0.04	0.07 ± 0.01
Leu	2.73 ± 0.06	0.04 ± 0.01
Lys	3.23 ± 0.05	
Met	0.75 ± 0.06	0.10 ± 0.01
Orn	0.17 ± 0.01	
Phe	1.83 ± 0.11	
Pro	10.10 ± 0.08	
Ser	3.03 ± 0.03	0.15 ± 0.04
Thr	2.47 ± 0.03	
Trp ^b^	–	
Tyr	0.79 ± 0.10	0.09 ± 0.07
Val	2.10 ± 0.02	0.14 ± 0.01
Hylys	1.04 ± 0.05	0.19 ± 0.05
Hypro	8.18 ± 0.11	

^a^ Composition and content of free and total amino acids in the hydrolysate were analyzed by using an amino acid analyzer. The data represent the mean ± SD of three experimental repeats; ^b^ Trp was not detectable because it was destroyed in the process of acid hydrolysis.

**Table 4:** Molecular weight distribution of peptides in the hydrolysate ^a^.

Molecular-Weight Range (Da)	Hydrolysate (%)
>10,000	9.68 ± 0.13
5000–10,000	6.25 ± 0.02
3000–5000	12.46 ± 0.11
1000–3000	16.49 ± 0.09
500–1000	15.60 ± 0.07
<500	39.50 ± 0.20

**^a^** The content of each range of peptides in the hydrolysate were calculated based on the percentage of the area of corresponding molecular weight range in the total chromatograph area of the hydrolysate in the HPLC chromatogram.

**Table 5:** Antioxidant activity towards DPPH• of collagen hydrolysates prepared from different collagen sources by different methods.

Antioxidant Activity	Hydrolysate Concentration (mg/mL)	Enzyme	Method	Collagen Source	Reference
32.8% ^a^	10	Aa2_1884 (S8)	Enzymolysis	Bovine bone	This study
40.7% ^a^	30	A69 (M4)	Enzymolysis	Bovine bone	[43]
341.91% ^a^	10	MCP-01 (S8)	Enzymolysis	Fish skin	[7]
50% ^a^	8.38		SWH ^b^	Fish bone	[44]
50% ^a^	7.58		SWH	Fish skin	[44]
50% ^a^	5.81	Pepsin	Enzymolysis	Fish skin	[45]
50% ^a^	1.57	Pepsin	Enzymolysis and fraction isolation	Fish skin	[46]

^a^ DPPH• scavenging ratio, ^b^ SWH, subcritical water hydrolysis.

## Data Availability

The amino acid sequence of Aa2_1884 has been submitted to NCBI database under the accession number WP_199608745, It can be found here: https://www.ncbi.nlm.nih.gov/protein/WP_199608745 (accessed on 6 December 2021).
